# Novel Therapeutic Targets in Melanoma

**DOI:** 10.3390/cancers15030747

**Published:** 2023-01-25

**Authors:** Michaël Cerezo, Stéphane Rocchi

**Affiliations:** INSERM, U1065, Centre Méditerranéen de Médecine Moléculaire (C3M), Université Côte d’Azur, Equipe 12, Bâtiment ARCHIMED, 151 Route de St Antoine de Ginestière, BP2 3194, CEDEX 3, 06204 Nice, France; michael.cerezo@univ-cotedazur.fr (M.C.); stephane.rocchi@univ-cotedazur.fr (S.R.)

Melanoma is the most aggressive skin cancer type and ranks amongst the deadliest cancers due to its ability to develop resistance to current therapies. In addition, it has a strong invasive capacity that allows the development of metastasis mainly in the lymph nodes, liver, lungs, and central nervous system. Over the past 15 years, targeted therapies have been developed against the mutated BRAF^V600^ (mutated in 50–60% of primary melanomas) and the MEK protein. The results of the combination of BRAF^V600^ inhibitors and MEK inhibitors have been revolutionary in the treatment of melanoma, with an increase in median progression-free survival (9.9 months for the combination versus 6.2 months for BRAF inhibitors alone) and tumor regression. However, after a few months of treatment, patients develop strong resistance to the therapies. In addition, melanoma cells can evade the immune response. This observation has led to the development of immune checkpoint inhibitors, allowing the reactivation of the immune system to eliminate melanoma cells. Currently, two types of immunotherapies are available in the form of blocking antibodies: Ipilimumab (anti-CTLA-4) and Nivolumab or Pembrolizumab (anti-PD-1). Ipilimumab targets the CTLA-4 receptor on CD4+ T cells, allowing their activation. This treatment increases survival rates in patients, but only 15% respond to this treatment. PD-1 is also expressed on T cells and its expression inhibits T-cell activation. Its target, PDL-1, is widely present in melanoma cells. Anti-PD-1 therapy elicits a response in approximately 30% of patients. Although these responses are objective and durable, approximately 65–70% of patients do not respond to this therapy. More recently, the combination of ipilimumab and nivolumab demonstrated increased efficacy compared to single agent treatment, but achieved at the expense of higher toxicity. Thus, even considering the major advances in melanoma treatment during the last ten or fifteen pasted years, we are now reaching a plateau with around fifty percent of patients responding to either therapy and a thirty percent overall survival rate for patients in the metastatic stage. To smash this glass ceiling, new therapeutic strategies should be developed for the melanoma cells themselves and the tumor microenvironment in order to target resistance mechanisms.

In this Special Issue, we present the state of the art in melanoma resistance and cover topics that include specific signaling pathways, epigenetic components, post-translational modifications, tumor cell dormancy, metastasis, and anti-tumor immunity.

To open this Special Issue, Datzmann and colleagues conducted a retrospective study based on a cohort of advanced malignant melanoma patients in Saxony to explore the differential responses to the different types of treatments [1]. Although a retrospective comparison between different treatment regimens is definitively complex, their study outlines the state of the art in melanoma treatment and highlights that the future of melanoma therapies involves combining multiple approaches/targets to bypass resistance. As a complement of this retrospective study, Comito et al. reviewed and summarized recently completed or ongoing clinical trials that have tested new strategies to fight melanoma [2].

To open new treatment perspectives, we selected a study from Raymond et al., that presented a comprehensive review of melanoma-associated GPCRs alterations and their consequences in terms of proliferation and aggressiveness, as well as in terms of their associated signaling pathway [3]. Indeed, with their prominent and pleotropic role in melanoma cell biology, GPCRs are attractive therapeutic targets. Nevertheless, as brilliantly presented in this review, this potential strategy faces several obstacles and the early stages clinical trials currently underway will arbitrate on the success or the failure of this strategy.

Next, considering that non-genetic reprogramming appears to be a major player in melanoma treatment adaptations fueling the emergence of resistance, we would like highlight three manuscripts that focus on the role of epigenetic and post-translational modifications in melanoma therapy. In their review, Gracia-Hernandez et al. explored the role of epigenetic alterations in immune evasion and therapy resistance [4]. They are particularly focused on the possible implication and therapeutic potential of epigenetic drugs to bypass resistance to targeted therapies and immune checkpoint inhibitors. Following this state-of-the-art overview, Mason et al. identified in their study, using an epigenetic targeted drug-screening, TP-472, a small molecule that inhibits bromodomain-7/9 (BRD7/9) and melanoma growth [5]. Mechanistically, they demonstrated that on top of inducing a pro-apoptotic signature, TP-472 downregulated several ECM proteins in melanoma cells. Considering that the upregulation of ECM proteins is a common mechanism in solid tumors that contributes to matrix stiffness, malignancy and metastasis, this work supports the clinical evaluation of TP-472 alone of more probably in combination with existing melanoma therapies. Ohanna and colleagues have chosen another attack angle and present an extensive review on the emerging role of deubiquitinating enzymes (DUBs) [6]. Interestingly, DUBs are involved in the pelorics aspect of tumorigenesis and aggressiveness, especially in melanoma, and could targeting existing pharmacological inhibitors to increase melanoma treatments and bypass resistance.

Broaching the topic of melanoma plasticity and metastasis, two of the main characteristics of melanoma cells, and explaining the aggressiveness and treatment failures of melanoma patients, Leo et al. report that Claisened Hexafluoro, a chemical analogue of Honokiol, inhibits melanoma cells spreading by blocking amoeboid-type motility and, in fine, melanoma metastasis capacities [7]. Mechanistically, authors have shown that Claisened Hexafluoro inhibits mitochondrial activity, leading to an activation of AMPK. This highlights the central role of energetic metabolism in the hallmarks of melanoma. Another characteristic of melanoma is its capacity to re-emerge months or even years after primary tumor resection. This mechanism, known commonly as dormancy, can explain the phenomenon whereby melanoma cells persist in a residual phase disease, without proliferation, but remain ready to awaken. In their review, Janowska and colleagues explore the genetic basis of melanoma dormancy/awakening and present a state of the art of possible therapeutic strategies that might thwart melanoma relapse, either by eliminating dormant cells or keeping them dormant [8].

Finally, we have selected two comprehensive reviews for inclusion that focus on alternative targets for immunotherapy. Reschke et al. present how innate immunity could be manipulated to transform a “cold” tumor microenvironment into “hot” one and improve the response to immune checkpoint blockage treatments [9]. As far as they are concerned, following the impressive results of the tebentafusp in uveal melanoma, Martinez-Perez and colleagues present a review that summarizes the evidence that targeting Gp-100 can be a gamechanger [10]. Interestingly, since Gp-100 is not only express in uveal melanoma, targeting Gp-100 could be also considered in the treatment of “classic” skin melanoma.

Taken together, this topic collection shows several options for the future development of alternative strategies against melanoma resistance. Given the high plasticity of melanoma cells and the multilevel complexity of resistance mechanisms, the future offers us enthusiastic prospects for identifying new therapeutic strategies.

We would like to thank all the authors, co-authors, and reviewers who accepted our invitation and contributed to this research topic, and we sincerely hope that the data and information conveyed within will be beneficial to the cancer research community.
